# Supplementary figures and images for: Fermented Rapeseed and Soybean Alone and in Combination with Macro Algae Inhibit Human and Pig Pathogenic Bacteria In Vitro
Source: Microorganisms. 2024 Apr 29;12(5):891. doi: 10.3390/microorganisms12050891 (PMC11124122; doi:10.3390/microorganisms12050891)

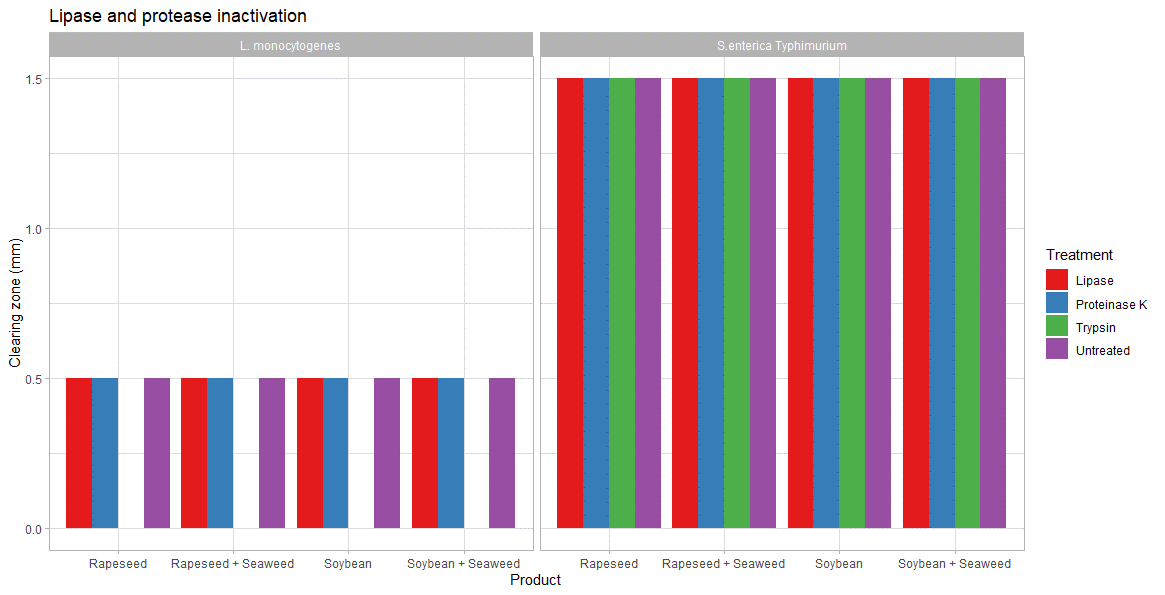

Supplement: Supplementary file 1 [file microorganisms-12-00891-s001.zip › microorganisms-2935063-supplementary.png]
